# Gelatine-Based Antioxidant Packaging Containing *Caesalpinia decapetala* and Tara as a Coating for Ground Beef Patties

**DOI:** 10.3390/antiox5020010

**Published:** 2016-03-31

**Authors:** María Gabriela Gallego, Michael H. Gordon, Francisco Segovia, María Pilar Almajano Pablos

**Affiliations:** 1 Chemical Engineering Department, Universitat Politècnica de Catalunya, Av. Diagonal 647, Barcelona 08028, Spain; maria.gabriela.gallego@upc.edu (M.G.G.); segoviafj@gmail.com (F.S.); 2 Department of Food and Nutritional Sciences, University of Reading, Whiteknights, P.O. Box 226, Reading RG6 6AP, UK; m.h.gordon@reading.ac.uk

**Keywords:** gelatine film, *Caesalpinia decapetala*, Tara, antioxidant activity, lipid oxidation, beef patties

## Abstract

The development of antioxidant-active packaging has numerous advantages, such as the reduction of synthetic additives in food, the reduction of plastic waste and food protection against oxidation reactions. Different concentrations of extracts of the plants *Caesalpinia decapetala* (CD) and *Caesalpinia spinosa* “Tara” (CS) were incorporated into gelatine films as natural antioxidants. The physical, mechanical and antioxidant properties of these films were studied. Films containing plant extracts at a high concentration had lower tensile strength with higher elongation at break points, compared to the control film (*p* < 0.05). Films exhibited antioxidant activity in the oxygen radical absorbance capacity (ORAC) and Trolox equivalence antioxidant capacity (TEAC) assays when added at 0.2%. The application of gelatine film containing CD and CS was found to be effective in delaying lipid oxidation and deterioration of beef patty quality during storage. Therefore, the films prepared in this study offered an alternative edible coating for the preservation of fresh food.

## 1. Introduction

Research into the development of edible films and coatings to extend the shelf-life of food products has attracted increasing interest in recent years due to several factors, such as consumer demand for high quality food [1], government demand for reducing packaging waste and marketing demand for new products. Additionally, the food industry is constantly seeking new and improved packaging systems and materials in a further attempt to retard deteriorative changes of food quality and, consequently, extend food shelf-life [2].

These films can help maintain and improve the quality of fresh, frozen and processed meat foods by reducing moisture loss, lipid oxidation and colour deterioration and acting as carriers for antimicrobial and antioxidant food additives [3].

Oxidation processes represent some of the most significant mechanisms that cause food spoilage. Lipid oxidation is one of the major limiting processes responsible for the reduction in food shelf-life, since it leads to off-flavour, off-odour and has been linked to oxidation reactions that cause product discoloration and loss of vitamins. Synthetic antioxidants have been used to prevent lipid oxidation, but the increasing demand for natural products has renewed the interest in natural polymers as raw materials for edible coatings or films due to their potential to extend the shelf-life of food and reduce the complexity and cost of packaging systems [4].

Traditional packaging technologies used for fresh meat and processed meat products have consisted chiefly of vacuum packaging, modified atmosphere packaging (MAP) and air-permeable packaging. In recent decades, technological advancements in materials, methodology and machinery have enhanced the efficiency and function of the packaging of meat products [5]. Gelatine is a protein-based polymer, widely used in the manufacture of edible films. As a consequence, the properties of films made from mammalian (chiefly porcine and bovine) gelatines have been widely studied [6]. Antioxidant active material is one type of “active packaging”, an innovative technology for food preservation based principally on mass transfer interactions between systems “food/packaging” [7]. The effect of active coating treatments with natural antioxidants on the uptake of lipid oxidation of food has been investigated [8]. Several studies have evaluated how antioxidants (Butylated hydroxytoluene (BHT), Butylated hydroxyanisole (BHA), alpha-tocopherol and natural extracts) incorporated in packaging film migrate out of the film and retard lipid oxidation in the stored foodstuff [9]. Different plant or herb extracts have been incorporated in gelatine films to enhance the antioxidant and/or antimicrobial properties, such as green tea extract [10,11], longan seeds and leaves [8], oregano or rosemary aqueous extracts [12] and murta ecotypes leaf extracts [13].

In order to meet consumer demands for more natural, disposable, potentially biodegradable and recyclable food packaging materials, research has focused on the incorporation of two plants rich in polyphenolic compounds, namely *Caesalpinia decapetala* (CD) and *Caesalpinia spinosa* (CS) (Tara), into the coating film. The genus *Caesalpinia* (Leguminosae) has long been used in Chinese traditional medicine. Plants of this genus have proven to be a rich source of compounds, such as diterpenoids, triterpenes, flavonoids, *etc.* However, information on their application to films is limited; thus, the aim of this work was to investigate the effectiveness of gelatine film to maintain the physicochemical properties of beef patties, maintaining the oxidative stability of these fresh food products.

Therefore, the objectives of this work were to develop a new type of active gelatine film enriched with Caesalpinia extract with added concentrations of ethanolic extract of CD and CS and to determine the lipid inhibition on ground beef patties during chilled storage, in addition to analysing the optical, mechanical, barrier and antioxidant properties of these films.

## 2. Materials and Methods

### 2.1. Plant Material

CD and CS plants were collected in spring 2015. Leaves were crushed and sieved using a sieve with a 40-micron mesh. Sieved leaves were stored inside a desiccator in the dark, at room temperature, until use.

### 2.2. Sample Extraction and Film Production

Extraction of the plants was done in 90 mL of EtOH:H_2_O adding either 6 g of the dried leaves of CD, 6 g of the dried leaves of white tea (WT) or 1.2 g of the dried leaves of CS (concentrations were obtained equal to the polyphenols between plants). For the extraction, the powder of the dried leaves of CD and CS was stirred using a magnetic stirrer with solvent for 24 h at 4 °C; then, the mixture was centrifuged at 2500 rpm, and the supernatant was collected.

Films were produced with Type A gelatine (2 g/100 g of filmogenic solution), which was hydrated (25 °C, 10 min) and then solubilized (55 °C, 15 min) in a thermostatic bath. After thorough solubilization, the plasticizing agent sorbitol (20 g/100 g of gelatine) was added, and the solution was magnetically stirred and the extract added. Three film formulations were made for each plant extract: CD1 (0.3%), CD2 (0.7%) and CD3 (1%) and CS1 (0.07%), CS2 (0.1%) and CS3 (0.2%). Similarly, two positive control films were prepared with BHA and WT. The film with BHA (synthetic antioxidant) was prepared at 0.001% and the film with WT (natural antioxidant) at 1%. Solutions were spread in polyethylene plates (60 g filmogenic solution/plate). The filmogenic solutions were dried in an oven at 30 °C, for 24 h.

### 2.3. Polyphenol Concentration and Activity in Films

The assay was performed according to Bodini *et al.* (2013) [14]. From these samples, analyses of total polyphenols by Folin-Ciocalteu and antioxidant activity by oxygen radical absorbance capacity (ORAC) and Trolox equivalence antioxidant capacity (TEAC) assays were performed [15].

### 2.4. Characterization of Bioactive Films

#### 2.4.1. Fourier Transform Infrared Spectroscopy

Analyses were carried out using a Brucker Vertex 70 Fourier Transform Infrared Spectroscopy (FTIR) spectrometer equipped with an attenuated total refection (ATR) accessory (Golden Gate, Specac Ltd., Orpington, UK), which is temperature controlled (a heated single-reflection diamond ATR crystal), was used. Films were directly placed in the beam, and eight scans were carried out in the spectrum range of 400 to 4000 cm^−1^.

#### 2.4.2. Mechanical Properties

Mechanical properties of the films were evaluated using a Zwick BZ2.5/TN1S (Zwick GmbH & Co. KG, ULM, Germany) testing machine, and measured with a deformation rate of 10 mm/min. Samples were cut off from regular films of a 15.2-µm thickness with a length of 10 mm and a width of 5 mm. The mechanical parameters were the average from a minimum of 10 measurements of each film sample. Tensile strength (*T*) and elongation (*E*) were obtained from the tension *vs.* elongation curves, and the elastic modulus (EM) was calculated.

#### 2.4.3. Water Vapour Permeability

Water vapour permeability (WVP) was determined according to [16] with some modifications. Films were fixed on the top of a glass permeation cell. The cell contained distilled water and was placed in a desiccator. These were placed in an incubator provided with temperature control (25 °C). The gain of mass in the films was determined for a period of 101 h.

#### 2.4.4. Light Transmission

The films were cut into a rectangle and placed in a spectrophotometer (Zuzi UV4200/51, AUXILAB, S.L., Navarra, Spain). According to the method proposed by Fang *et al.* (2002) [17], the films were measured at 600 nm in triplicate. The opacity was calculated based on the following equation:

(1) Opacity=Abs600x

where *Abs*_600_ is the value of absorbance (*Abs*) at 600 nm and *x* is the film thickness (mm).

#### 2.4.5. Scanning Electron Microscopy

The morphology of the film was examined by scanning electron microscopy (Nova Nano SEM 230, FEI, Hillsboro, OR, USA). Films were cut into 5 mm × 5 mm pieces. Samples were gold coated and observed using an accelerating voltage of 2 0 kV.

#### 2.4.6. Colour Properties

The colour parameters lightness (L*), redness (a*) and yellowness (b*) were measured using a Konica Minolta CM-3500d colorimeter (Konica Minolta Sensing, INC., Milton Keynes, UK). Measurements were taken in different film portions. The values reported are the average of 9 measurements.

### 2.5. Evaluation of Antioxidant Activity in Food

#### 2.5.1. Preparation of Beef Patties

Fresh beef was purchased from a food market. The meat was mixed with salt (1.5%). The burgers were formed manually, and five formulations were prepared: negative control (without antioxidant), CD3 (film with CD3 extract), CS3 (film with CS3 extract), BHA (film with BHA) and WT (film with WT extract). The films were placed on the top and bottom of the hamburger.

#### 2.5.2. TBARS Assay

The thiobarbituric acid reactive substances (TBARS) method was used to measure the lipid oxidation as described by Gallego *et al.* (2015) [18].

#### 2.5.3. Metmyoglobin

Five grams of patty samples were homogenized with 25 mL of 0.04 M phosphate buffer (pH 6.8) for 10 s using an Ultra-Turrax mixer (IKA, Staufen, Germany). After 1 h at 4 °C, the homogenate was centrifuged at 4500× *g* for 20 min at 4 °C. The absorbance of the filtered supernatant was read at 572, 565, 545 and 525 nm. The percentage of metmyoglobin was calculated as follows:

(2) $$MetMb\;\left(\%\right)=\left[2.514\;\left(A_{572}/A_{525}\right)+0.777\;\left(A_{565}/A_{525}\right)+0.8\;\left(A_{545}/A_{525}\right)+1.098\right]\times 100$$

## 3. Results

### 3.1. Total Phenolic Content and Antioxidant Activity

**Figure 1:**
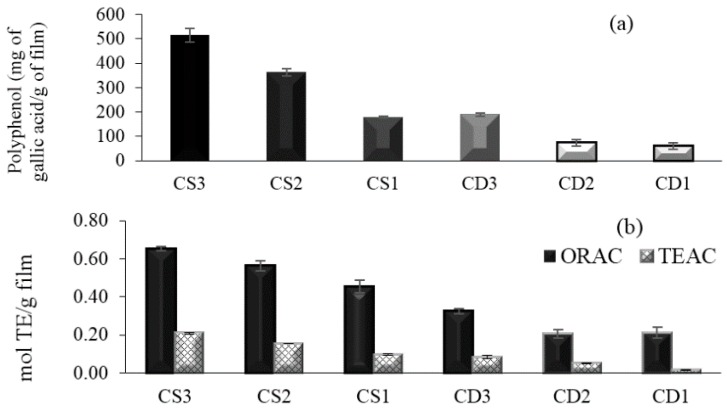

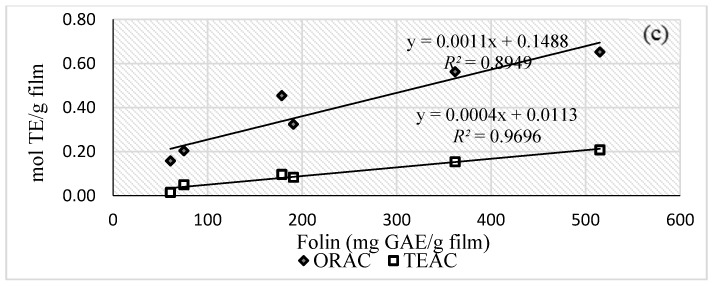
(**a**) Polyphenol concentration of films with different concentrations of plants extracts: *Caesalpinia spinosa* 1 (CS1) (0.07%), CS2 (0.1%), CS1 (0.2%), *Caesalpinia decapetala* 1 (CD1) (0.3%), CD2 (0.7%), CD1 (1%); (**b**) Antioxidant activity by oxygen radical absorbance capacity (ORAC) and Trolox equivalence antioxidant capacity (TEAC) assays of films with different concentrations of plant extracts; (**c**) Correlation for polyphenol content from the Folin assay with antioxidant activity assessed by the TEAC and ORAC assays.

Folin-Ciocalteu phenol reagent was used to obtain an estimate of the phenolic groups present in the gelatine film containing plant extracts. The control films (without extract) contained no phenolics (data not shown). The total polyphenol content of gelatine film with herb extracts is shown in Figure 1a. As expected, total phenol content increased significantly by the incorporation of more extract (*p* < 0.05). The phenol content as gallic acid equivalent (GAE) per gram of films ranged from 178 to 515 mg GAE/g film for CS and 61 to 191 mg GAE/g film for CD. The highest value (515 ± 27 mg GAE/g film) was for the film formulated with CS3 (0.2% extract), and the lowest value (61 ± 12 mg GAE/g film) was for the film with CD1 (0.3% extract).

The antioxidant activity was measured by the ORAC and TEAC assay (Figure 1b). The ORAC method measure the loss of fluorescence of a probe (fluorescein) in the presence or absence of an antioxidant. The ORAC value was 0.65 ± 0.01 mol Trolox Equivalent (TE)/g film for CS3 at a concentration of 0.2% and 0.32 mol TE/g for CD3 at a concentration of 1%. Moreover, when using the TEAC assay, based on the ability of an antioxidant to reduce the ABTS^•+^ radical [19], the antioxidant activity was higher at 0.21 ± 0.007 mol TE/g film for CS3 and lower at 0.02 ± 0.001 mol TE/g film for CD3.

The polyphenol content assessed by the Folin-Ciocalteu assay correlated with antioxidant capacity assessed by the TEAC and ORAC assays (Figure 1c). The TEAC assay was the antioxidant assay that best correlated with the total phenolic content in the extracts (*R^2^* = 0.9696). The correlation coefficient for the ORAC assay was 0.8949.

### 3.2. Characterization of Bioactive Films

#### 3.2.1. Fourier Transform Infrared Spectroscopy

**Figure 2:**
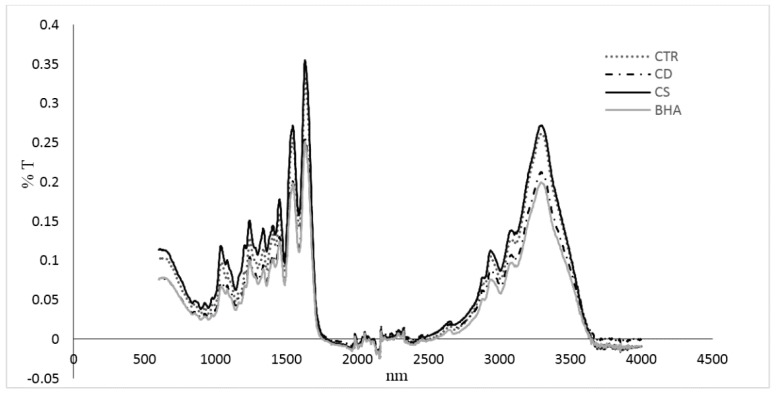
Infrared spectra of gelatine-based films treated with different samples: CTR (control), BHA, CS3 and CD3.

The FTIR spectra of films are shown in Figure 2. Samples showed the amide A band located at about 3300 cm^−1^, the amide I band located between 1700 and 1600 cm^−1^, the amide II band located between 1600 and 1500 cm^−1^ and the amide III band between 1200 and 1400 cm^−1^.

#### 3.2.2. Mechanical Properties

The effects of CD and CS addition on the tensile strength (TS) and elongation at break(EAB) of the film are presented in Table 1. The addition of CD1 (0.3%), CS1 (0.07%) and CS2 (0.1%) significantly increased tensile strength. CD2, CD3 and CS3 samples had values below that of the control sample. It was observed that values of EAB were inversely proportional to the concentration of the plant extracts added in the film, showing a decrease by increasing the concentration of the plants, and in the case of TS, it was directly proportional to the concentration of plant extracts.

#### 3.2.3. Water Vapour Permeability

WVP is one of the most important parameters for biodegradable films. This parameter was studied to evaluate the combined effect of CD and CS on the barrier properties of the gelatine film. The WVP of the gelatine-based film containing CD3 (1%), CS3 (0.2%) and BHA is shown in Table 2 As can be seen from this table, the control film showed the highest WVP, followed by the film containing added CS. The lowest WVP was found in the gelatine film treated with CD and BHA. There were no significant differences between the CD film and the BHA-treated film (*p* > 0.05).

#### 3.2.4. Light Absorption

Table 3 shows the absorption of samples at 600 nm and the opacity. The lowest transmittance was for the film containing BHA. The film with CD3 had the lowest percent transmittance (%*T*) for UV radiation (64.86%), and the opacity value was higher (14.46%), compared to the control, so it is clear that inclusion of the extract at this concentration into gelatine improved the light barrier properties.

#### 3.2.5. Scanning Electron Microscopy

**Figure 3:**
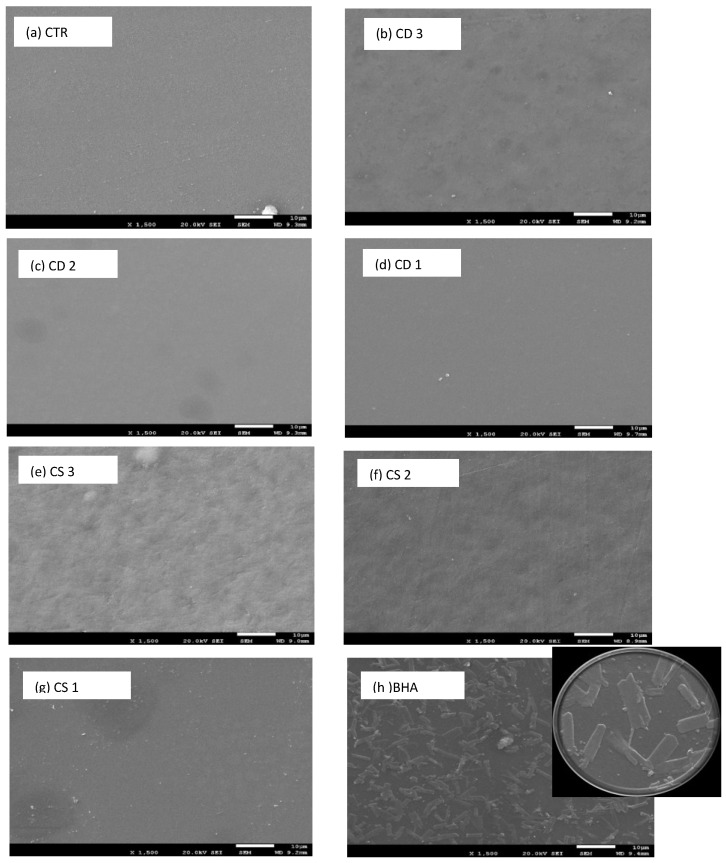
Scanning electron microscopy of gelatine films with different concentrations of plant extracts (1500×).

Figure 3 shows the Scanning Electron Microscopy (SEM) of gelatine films treated with CD, CS and BHA. It was observed that the surface of the control film was very homogeneous without bubbles. The incorporation of the synthetic antioxidant BHA allowed the formation of crystals in the film matrix. The addition of more plant extracts to the film generated a more heterogeneous surface. In the case of film containing CD1 and CD2 extracts, the samples showed an appearance very similar to the control, and the same was observed in the film treated with CS1 Tara extract.

#### 3.2.6. Colour Properties

In practical applications, colour quality might influence the appearance of edible films, which in turn affects the acceptance of foods by consumers. The incorporation of plant extracts improves the film’s ability to block UV and visible light. The *Commission Internationale de l'Éclairage* (in French, (CIE) colour values in the films with or without herb extracts are shown in Table 4. L* values did not differ significantly between samples. For example, the L* values for control, CD film and CS film were 74.12 ± 1.21, 72.35 ± 1.04 and 75.16 ± 0.81, respectively. With the addition of phenolic compounds, the L* values of composite films were practically unchanged. The control had an initial yellowness (b*) value of 4.51 ± 1.47, and the addition of the extracts caused a significant increase (*p* < 0.05) in this value.

### 3.3. Evaluation of Antioxidant Activity in Food

#### 3.3.1. TBARS Assay

**Figure 4:**
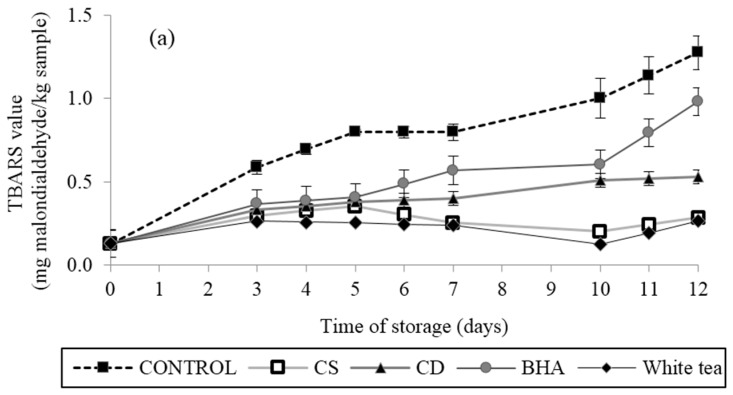

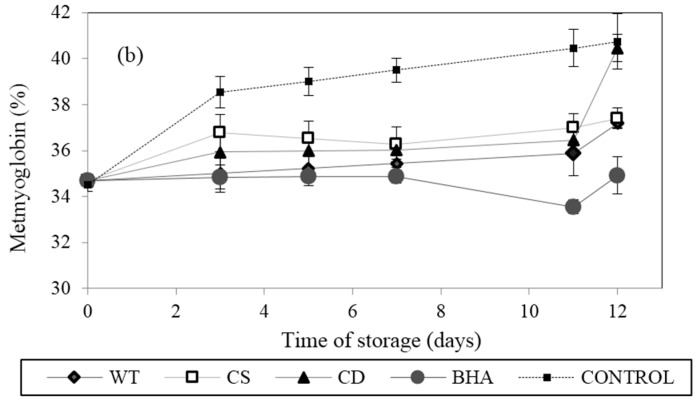
(**a**) Effects of films with CD and CS added at 1% and 0.2%, respectively, on the TBARS value (mg of malondialdehyde equivalent/kg of sample) of raw beef patties during 12 days of refrigerated storage at 4 °C and (**b**) effects of CD and CS extract added on metmyoglobin changes in beef patties during 12 days of refrigerated storage at 4 °C. Results are given as the mean ± standard error.

Fat content is one of the most important quality indicators of minced beef products. The minced beef has approximately 17.3% of fat [20]. A direct method (TBARS) was used to evaluate the effectiveness of the antioxidants in preventing oxidative degradation of lipids with the production of compounds, such as conjugated hydroperoxides and aldehydes. The TBA values were calculated as mg MDA (Malondialdehyde)/kg meat. TBARS indices were significantly (*p* < 0.001) affected by storage time and the active packaging system. The TBARS values of all samples increased continuously up to 12 days of storage (Figure 4a). TBARS values of control, CD3 (1%), CS3 (0.2%), WT (1%) and BHA were 0.59 ± 0.04, 0.33 ± 0.01, 0.30 ± 0.00 and 0.26 ± 0.01 mg MDA/kg at Day 3 and increased to 1.27 ± 0.10, 0.53 ± 0.01, 0.27 ± 0.02, 0.27 ± 0.04 and 0.98 ± 0.01 mg MDA/kg at the end of storage (Day 12). The highest rate of increase was observed in the patties treated with the control gelatine film (without plant extract), while the lowest TBARS value was found in the patties stored with gelatine film treated with CS, followed by samples treated with WT.

#### 3.3.2. Metmyoglobin

The rates of formation of surface metmyoglobin in fresh beef patties are shown in Figure 4b. Metmyoglobin percentage for the control increased rapidly in the first seven days of storage, reaching values above 39.5%, but in samples stored under films treated with antioxidants, the increase in TBARS values was slow and steady.

## 4. Discussion

### 4.1. Total Phenolic Content and Antioxidant Activity

Packaging with antioxidant properties is a promising technique to extend the shelf-life and maintain the quality of food. The Folin assay is a useful tool to know the total polyphenols, and the ORAC and TEAC assays are important techniques for measuring antioxidant activity.

The Folin values were higher than those for other films containing natural extracts with antioxidant power, as the film incorporated with aqueous chitosan green tea extract (20% *w*/*v*), where the total polyphenol content was about 33 mg gallic acid/g film [21]. Similarly, according to the study by Araujo *et al.* 2015 [22] with ethanolic extract propolis, the polyphenols were proportional to the extract incorporated in films. The antioxidant capacity of the films should be strongly related to the portion of film that can be dissolved in water and, consequently, to the release of active compounds.

The antioxidant capacity assessed by the ORAC and TEAC assays was higher in the CS3 (0.2%) extract films. The antioxidant activity is in direct relation to the concentration of polyphenols in the herb extract. Comparing to other plants, the ORAC value for the film containing CS was higher than the values found for commonly-consumed herbs with high antioxidant capacity, including basil, marjoram, oregano, ginger, thyme and black tea (0.048 mmol/g dry plant, 0.27 mmol/g dry plant, 0.14 mmol/g dry plant, 0.39 mmol/g dry plant, 0.27 and 0.013 mmol/g dry plant, respectively). The results obtained for these assays are consistent with the CS composition, which contains a high proportion of phenolic compounds. The phenolic compounds are free radical acceptors that delay or inhibit the initiation step of autoxidation or interrupt the autoxidation propagation step [23]. The high antioxidant capacity is due to the rich content of polyphenols contained in these plants, especially CS. The CS tree was traditionally considered the second-richest tannin feed stock after Schinopsis balansae [24]. Many studies over recent years have demonstrated that the antioxidant activity of plants is caused mainly by phenolic compounds [25].

The results indicated that incorporation of CS and CD into gelatine film enhanced the antioxidant activity of the film. Several studies show that the incorporation the ethanolic extracts of plants improve the antioxidant power of film. Norajit *et al.* (2010) [26] observed that the incorporation of a ginseng ethanolic extract improved the antioxidant activity of film compared to the control film. Gómez-Estaca *et al.*, 2009 [12] showed that the incorporation of borage ethanolic extract to gelatine films gave rise to a high reducing ability with FRAP.

### 4.2. Characterization of Bioactive Films

#### 4.2.1. Fourier Transform Infrared Spectroscopy

FTIR spectra showed that there was no interaction between the functional groups of plants and gelatine. The absorption bands in the spectra were situated in the amide band region. Amide A is attributed to the stretching of the N–H group. The amide I band is related to the stretching CO, and the amide II band is related to the stretching of C–N and the angular distortion of the N–H bond. The samples containing CD and CS showed similar spectra to the control gelatine film, suggesting that there is no interaction between the functional groups of the extracts and gelatine. Similar results are in agreement with [14,27].

#### 4.2.2. Mechanical Properties

Different antioxidant agents can influence the mechanical properties of the films. Our results for mechanical properties are consistent with various other studies. Bodini *et al.* (2013) [14] in their study with gelatine films using propolis extract suggested that the ethanol propolis extract acted as a plasticizing agent, increasing the mobility of the polymer matrix, which, in turn, promoted a reduction in tensile strength and increased film elongation. Nuñez *et al.* (2013) [28] reported that the addition of lignin produced an evident plasticizing effect, as deduced from significant decreases in TS in the composite films, alongside a marked increase in EAB. Hoque *et al.* (2011) [29] studied the mechanical properties of films prepared from gelatine and partially hydrolysed gelatine containing different herb extracts. Films made from gelatine containing cinnamon, clove and anise showed higher TS, but lower EAB. Lim *et al.* (2010) [30] reported that the TS of agar-based films containing nano-clays decreased with an increased concentration of grape seed extract in the film matrix up to 1.2%. Similar results have been also reported for agar-based films containing green tea extracts [10].

In general, the incorporation of polyphenol-rich aqueous extracts reduces the mechanical properties of the films [10]. However, there is a relationship between the concentration of the plant extract and the film’s mechanical properties.

#### 4.2.3. Water Vapour Permeability

The results obtained showed that the addition of CS extract improved the barrier properties of gelatine films. Rattaya *et al.* (2009) [31] said that the chemical nature of the macromolecule, the structural/morphological characteristics of the polymeric matrix, the chemical nature of the additives, as well as the degree of cross-linking all affect the barrier characteristics of the film. Wu *et al.* (2013) [11] reported that the incorporation of green tea extract into gelatine film caused the resulting film to have lower WVP. They hypothesized that polyphenolic compounds could fit into the gelatine matrix and establish cross-links with the reactive groups of the gelatine through hydrogen bonds or through hydrophobic interactions. Bodini *et al.* (2013) [14] reported that the incorporation of ethanol-propolis extract led to a significant reduction in WVP in relation to the control gelatine film. The WVP should be as low as possible for food packaging in order to avoid or at least to reduce moisture transfer between the food and the atmosphere, or between two components within a heterogeneous food product [32].

#### 4.2.4. Light Absorption

Transparent packaging allows the oxidation and degradation of nutritional compounds, because light acts as a catalyst for these processes. Therefore, opaque packaging and packaging containing specific compounds that absorb light in the UV-Vis spectrum have been developed to prevent these reactions. Plant extracts are commonly used to provide colour and opacity to polymers. A food packaging film is required to protect food from the effects of light, especially UV radiation [25].

In general, light transmission at 600 nm for all films was in the range of 64.86%–87%. With the addition of herb extracts, variations in light transmission of the resulting films were observed. The incorporation of a higher concentration of the extracts in the film produces an increase in opacity of the film. The observed differences in the transparency of the films can be attributed to differences in concentration, colour, polyphenols present in extracts and the extracts’ interaction with the gelatine film [27].

#### 4.2.5. Scanning Electron Microscopy

Higher concentrations of the extracts used in the films (CD3, CS2 and CS3) formed a more heterogeneous structure with pore formation in the matrix. The formation of a heterogeneous surface can be related to a reduction of the dissolution of extract in gelatine when the concentration is higher. Similar results have been reported by other authors. Bodini *et al.* (2013) [14] showed that increased ethanol-propolis extract (EPE) concentrations produced an increase in the porosity of the matrix, which was probably associated with EPE distribution in the polymer matrix. Hoque *et al.* (2011) [29] showed that a smooth surface was also obtained in the film prepared from gelatine treated with star anise. Furthermore, Li *et al.* (2014) [25] reported that the micrographs of films displayed a heterogeneous surface and porous appearance after the addition of natural antioxidants (grape seed extract, gingko leaf extract, green tea extract) at a concentration of 1.0 mg/mL. In general, the smooth surface became rougher when the concentration of herb extracts was increased.

#### 4.2.6. Colour Properties

As a general trend, the addition of phenolic compounds caused an increase of blueness for composite films as indicated by increased b* values and caused a decrease of redness (a* values), except for the CS film.

Previous authors have reported that the addition of phenolic compounds caused a reduction of the brightness of edible films [33]. Ahmad *et al.* (2012) [34] showed that the incorporation of lemongrass oil into fish skin gelatine films increased its total colour difference. However, no marked effect on the colour parameters of films was obtained when ginger oil was added [35]. The type of plant used influenced the colour of the gelatine film, depending on the type and concentration incorporated.

### 4.3. Evaluation of Antioxidant Activity in Food

#### 4.3.1. TBARS Assay

It was noted that the films containing natural extracts did not reach a value of 1.5 mg MDA/kg by the end of the long storage period (Day 12). It has been reported that an index of 1.5 is closely related to perceptible and unacceptable off-odour of meat [36].

In our study, samples stored with WT film and CS film behaved similarly with no significant difference (*p* > 0.05). However, when compared to the sample stored under CD film, there was a noticeable difference, since this was less effective. In this study, the samples reduced lipid oxidation relative to the control in the following order: CS > WT > CD > BHA. These results are consistent with polyphenol concentrations obtained with the Folin method, which gave values for CD (27.71 ± 1.03 mg GAE/g dry plant) and CS (368.90 ± 0.6 mg GAE/g dry plant) and with antioxidant activity assessed by the ORAC method that showed the same order for the plant extracts.

The mechanism by which the antioxidant-treated packaging lowers the number of molecules reactive to TBA is currently under investigation. Inactivation of free radicals by either migration of antioxidant molecules from the active film to the meat or scavenging of those oxidant molecules from the meat into the active film may be considered as hypotheses for its mechanism of action. Our results are in agreement with various other studies in films, all of which reported that natural antioxidants from culinary herbs and edible plants were effective at controlling lipid oxidation and extending the shelf life of meat products. However, Lorenzo *et al.* (2014) [37] studied the effect of antioxidant active systems on lipid stability of foal steaks during storage. Active films with oregano essential oil (2%) resulted in a decreased lipid oxidation of foal steaks, and these were more efficient than those treated with green tea (1%). Similarly, Camo *et al.* (2008) [38] reported that fresh lamb steaks were treated with three different preparations of natural antioxidants containing rosemary and oregano, which resulted in the enhanced oxidative stability of lamb steaks. Furthermore, similar results have been obtained when other compounds were incorporated, such as essential oils [34] and grapefruit extracts [39]. In all cases, the oxidation rates decreased, maintaining an acceptable quality in meat, poultry or fish products.

These results indicated that lipid oxidation in beef patties could be minimized by the use of gelatine film treated with natural extracts. We found that a concentration of 0.2% of CS extract in the film showed strong antioxidant activity against lipid oxidation in ground beef patties.

#### 4.3.2. Metmyoglobin

The discoloration that occurs during the display of red meat cuts is generally associated with the accumulation of metmyoglobin in the meat surface. During refrigerated storage, MetMb accumulation and meat discoloration largely depend on the presence of reducing systems in meat and on lipid oxidation [40].

Djenane *et al.* (2002) [41] reported that a consumer panel rejected samples of fresh beef with a percentage of MetMb greater than 40%. Using this limit, the presence of natural antioxidant extracts led to a significant (*p* < 0.05) inhibition of metmyoglobin formation. The film treated with CS reduced the % of metmyoglobin (37.40%) to less than 40%, and this value was very similar to that obtained in treatment with WT (37.19%), showing the positive effect of the natural extracts in the active films on the colour of refrigerated fresh patties.

## 5. Conclusions

From the results presented, it is possible to conclude that CD and CS incorporation caused an improvement of the gelatine film properties, reducing tensile strength, and the CS extract reduced the permeability to water vapour of films relative to the control. Films exhibited high antioxidant activity, especially for films prepared with the addition of 0.2% of CS and 1% of CD. The analysis of TBARS provided a complete assessment of the consequences of lipid oxidation in beef patties. The CS film was the most effective antioxidant for the beef patties, inhibiting the formation of TBARS more effectively than the synthetic antioxidant BHA over the course of 12 days. Results indicated that biodegradable gelatine films containing CD and CS have good potential for utilization in food packaging. Advances in active packaging materials based on renewable sources, such as gelatine containing natural extracts, will open new lines of research for the development of improved eco-friendly materials.

## Figures and Tables

**Table 1:** The effects of CD and Tara at different concentrations on the tensile strength (TS) and elongation at break (EAB) of gelatine films.

Sample	TS (Mpa)	EAB (%)
CTR	69.8 ± 9.5 ^a,c^	95.6 ± 13.5 ^a^
CD 1	96.4 ± 6.2 ^b^	108.0 ± 15.9 ^a^
CD 2	68.5 ± 18.2 ^a^	231.1 ± 82.6 ^b^
CD 3	57.0 ± 5.8 ^c^	404.4 ± 51.8 ^c^
CS 1	123.8 ± 1.5 ^d^	178.9 ± 53.3 ^b^
CS 2	86.6 ± 10.1 ^a^	58.6 ± 5.8 ^d^
CS 3	55.7 ± 10.6 ^c^	357.4 ± 40.1 ^c^
BHA	109.5 ± 12.5 ^e^	62.9 ± 15.2 ^d^

Different lowercase letters (^a–e^) in the same column indicate significant differences (*p* < 0.05) between samples.

**Table 2:** Water vapour permeability (WVP) of the different gelatine films.

Sample	WVP
Control	1.94 ^a^ ± 0.1
CD 3	1.11 ^b^ ± 0.2
CS 3	1.32 ^c^ ± 0.2
BHA	1.12 ^b^ ± 0.1

Different lowercase letters (^a–c^) in the same column indicate significant differences (*p* < 0.05) between samples.

**Table 3:** Light transmission (%*T*) and opacity of gelatine films with CD and CS extracts at different concentrations of the different gelatine films.

Samples	%*T*	Opacity
Control	87.00	4.46 ^a^ ± 0.02
CD 1	86.30	4.92 ^d^ ± 0.02
CD 2	85.51	5.23 ^c^ ± 0.03
CD 3	64.86	14.46 ^b^ ± 0.01
CS 1	88.10	4.23 ^g^ ± 0.02
CS 2	87.30	4.54 ^f^ ± 0.03
CS 3	85.90	5.08 ^e^ ± 0.02
BHA	79.98	7.46 ^h^ ± 0.01

Different lowercase letters (^a–h^) in the same column indicate significant differences (*p* < 0.05) between samples.

**Table 4:** Colour parameters of gelatine films: control, CD3 (1%), CS3 (0.2%) and BHA.

Samples	*L* *	*a* *	*b* *
**Control**	74.12 ^a^ ± 1.21	0.86 ^a^ ± 0.30	4.51 ^a^ ± 1.47
**CD 3**	72.35 ^b^ ± 1.04	−1.39 ^b^ ± 0.63	8.37 ^b^ ± 1.45
**CS 3**	75.16 ^a^ ± 0.81	2.33 ^c^ ± 1.33	8.15 ^b^ ± 1.52
**BHA**	73.93 ^a^ ± 1.53	−1.69 ^d^ ± 0.41	7.77 ^b^ ± 0.80

Mean values are showed for color parameters (lightness, *L* *; redness, *a* *; yellowness, *b* *). Different lowercase letters (^a–d^) in the same column indicate significant differences (*p* < 0.05) between samples.

## Abbreviations

The following abbreviations are used in this manuscript:

CD: *Caesalpinia decapetala*
CS: *Caesalpinia spinosa*
FTIR: Fourier transform infrared spectroscopy
*T*: Tensile strength
EM: Elastic modulus
WVP: Water vapour permeability
SEM: Scanning electron microscopy
MetMb: Metmyoglobin
